# Supplementary material for: The interactome of intact mitochondria by cross-linking mass spectrometry provides evidence for coexisting respiratory supercomplexes
Source: Mol Cell Proteomics. 2017 Dec 8;17(2):216–32. doi: 10.1074/mcp.RA117.000470 (PMC5795388; doi:10.1074/mcp.RA117.000470)
Supplement: Supplemental Data [file supp_RA117.000470_133922_0_supp_23978_fzffwf.zip › spectra_annotation/mito_DR_spectra_annotation/367-1-9-1-71-1.pdf]

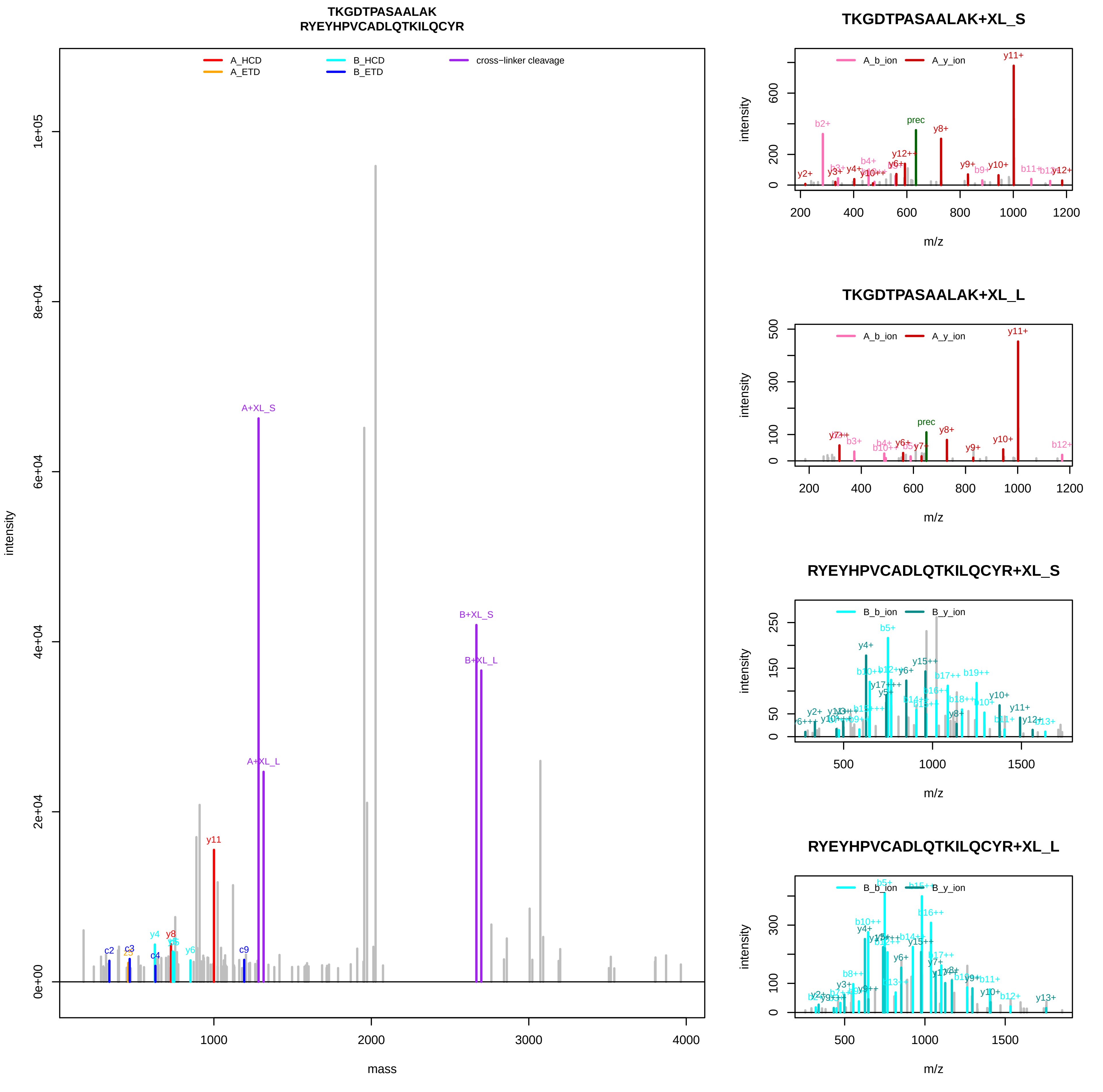

TKGDTPASAALAK
RYEYHPVCADLQTKILQCYR
A_HCD
A_ETD
B_HCD
B_ETD
cross−linker cleavage
A+XL_S
A+XL_L
B+XL_S
B+XL_L
y11
y8
y4
y5
y6
c2
c3
z3
c4
c9
intensity
mass
0e+00
2e+04
4e+04
6e+04
8e+04
1e+05
1000
2000
3000
4000
TKGDTPASAALAK+XL_S
A_b_ion
A_y_ion
prec
y11+
y8+
b2+
y12++
y9+
y10+
y2+
y3+
y4+
b4+
y6+
b11+
y12+
intensity
m/z
TKGDTPASAALAK+XL_L
A_b_ion
A_y_ion
y11+
prec
y8+
y10+
y9+
b3+
b4+
y6+
y7+
b12+
intensity
m/z
RYEYHPVCADLQTKILQCYR+XL_S
B_b_ion
B_y_ion
b5+
y4+
y15++
y6+
b17++
b19++
y10+
y11+
y12+
b13+
y2+
y5+
y8+
intensity
m/z
RYEYHPVCADLQTKILQCYR+XL_L
B_b_ion
B_y_ion
b5+
b16++
b10++
y4+
y15++
y6+
b17++
y7+
b8++
y9+
b11+
y10+
b12+
y13+
intensity
m/z
